# Finite element analysis of medial meniscus tears and related surgical techniques with biomechanical validation

**DOI:** 10.3389/fbioe.2026.1916206

**Published:** 2026-09-09

**Authors:** Chengyue Yu, Kexin Liu, Jingyu Zhang, Wenjun Zhao, Xiaoyuan Duan, Bo Liu, Lupeng Wang, Zhao Liu, Yongxin Huo, Tao Zhang, Guosheng Xing, Weiguo Xu

**Affiliations:** 1 Tianjin Hospital, Tianjin University, Tianjin, China; 2 Weifang People’s Hospital, Weifang, Shandong, China; 3 Changchun Institute of Optics, Fine Mechanics and Physics, Chinese Academy of Sciences, Jilin, China; 4 The Second Hospital of Tangshan, Tangshan, Hebei, China

**Keywords:** longitudinal tear, medial meniscus, meniscectomy, meniscus repair, radial tear

## Abstract

**Introduction:**

This study established finite element models of the knee incorporating radial and longitudinal meniscal tears and simulated corresponding surgical techniques to investigate the influence of different tear types, lengths, locations on stress distribution in the knee joint and evaluate the restorative effects of different surgical techniques on the biomechanical performance of the meniscus.

**Methods:**

A three-dimensional finite element model of the right knee including bone and soft tissues was developed. Stable and unstable radial and longitudinal tear models were created in the posterior horn of the meniscus across three zones (white-white, red-white, and red-red zones), together with the corresponding surgical technique models (suture repair and partial meniscectomy). Finite element analysis was performed under static loading. A cadaveric knee biomechanical experiment was also conducted to validate the finite element model.

**Results:**

Under static loading, the discrepancy in medial compartment contact area between the finite element model and the cadaveric experiment under intact meniscus conditions was 5.72%. The peak contact pressure was highest in the unstable radial tear model, reaching 14.88 MPa, representing an 181.82% increase compared with 5.28 MPa measured in the intact meniscus. Both repair and partial meniscectomy significantly reduced the peak contact pressure in the medial meniscus and tibial cartilage for radial tears. Repair reduced the peak pressure by 35.35% for stable radial tears, while partial meniscectomy reduced it by 49.60% for unstable radial tears. Meniscal repair reduced the peak contact pressure by 4.55% and 3.85% for stable longitudinal tears located in the red and white zones, respectively, and restored hoop stress transmission. Partial meniscectomy reduced the peak contact pressure by 7.90% (0.67 MPa) for unstable longitudinal tears located in the white zone.

**Conclusion:**

Stress distribution in the meniscus and its components is influenced by tear types, locations, lengths, and surgical technique. Surgical outcomes significantly depend on tear types, lengths, and locations. The extent of biomechanical restoration varies considerably among different surgical techniques, suggesting that clinical procedure selection should be based on personalized consideration of injury characteristics.

## 1 Introduction

The knee joint, the largest and most complex joint in the human body, plays a pivotal role in supporting daily activities such as walking and running (Yu et al., 2025). The meniscus plays a critical role in load transmission, shock absorption, and protection of the articular cartilage within the knee joint (Wang J. Y. et al., 2021; Li et al., 2022; Zhao et al., 2024). Due to the high mechanical load in daily activities, meniscus tears are easily induced, which can further lead to abnormal wear and tear of articular cartilage, thereby accelerating the pathological progression of osteoarthritis (OA) (Liu et al., 2022).

Finite element analysis plays an important role in orthopedic research and has been widely applied in areas such as material mechanical property evaluation, orthopedic internal fixation device design, and joint contact mechanics analysis (Inal et al., 2018; Gok et al., 2019; Inal et al., 2019; Gok and Gok, 2024; Kurt et al., 2025a; Kurt et al., 2025c). It has also been successfully employed to reveal the biomechanical effects of meniscal injuries and the mechanical mechanisms of corresponding surgical techniques. With a clearer understanding of the function of the intact meniscus and the degenerative changes that occur following meniscectomy, the treatment philosophy for meniscal tears has undergone a fundamental shift from total or subtotal meniscectomy toward reparative surgery that preserves as much healthy tissue as possible (Steineman et al., 2022; Chen et al., 2023; Knapik et al., 2025; Ma et al., 2025; Yan et al., 2025). Ma et al. (2025) investigated the effects of medial meniscus radial tears and subsequent surgical treatments (repair or meniscectomy) on knee joint mechanics during normal walking. Their findings demonstrated that meniscal repair can significantly restore the original function of the meniscus. The finite element study by Rivarola et al. (2026) showed that meniscal repair can restore stress distributions close to physiological levels. Xu et al. (2022) examined partial meniscectomy and repair for complete radial and oblique tears of the meniscal posterior root. Their results indicated that meniscal repair exerts a positive effect on restoring the normal biomechanical properties of the medial meniscus. Wang et al. (2021) demonstrated that posterior root suture repair can significantly improve joint contact pressure and contact stress. Chen et al. (2023) developed a finite element model of horizontal meniscal tears and investigated the optimal surgical strategy. Their results showed that suture repair is the best method for maintaining knee joint force balance. However, when suture repair is difficult, resection of the superior meniscal leaf also represents a reliable option. Similarly, Yan et al. (2025) studied the progressive healing process of horizontal meniscal tears under flexed conditions. Their findings revealed that the mechanical function of the meniscus gradually recovers as healing progresses. In a recent study, Kurt et al. (2026) performed finite element analysis on radial, horizontal, and complex meniscal tears, demonstrating that radial tears significantly alter knee joint biomechanics and increase tissue stress. Furthermore, they utilized finite element analysis to evaluate an alternative surgical technique for repairing meniscal root tears. Their results indicated that this approach optimizes biomechanical stability and promotes more favorable healing conditions (Kurt et al., 2025b).

Clinical and biomechanical studies have shown that the specific characteristics of a meniscal tear, including the tear pattern (such as radial, longitudinal tears), tear length, and location (red-red zone, red-white zone, or white-white zone), are important parameters affecting the feasibility of repair, the choice of surgical procedure, and postoperative efficacy (Kedgley et al., 2019). Currently, meniscal-preserving surgery falls into two main categories: partial meniscectomy (partial resection) and meniscal repair (suture). Over the past 3 decades, meniscal repair has emerged as the preferred treatment option, owing to its superior ability to restore native biomechanical function (Berk et al., 2022; Mameri et al., 2023; Wei et al., 2023; Ina et al., 2025). Although these surgical approaches yield varying prognostic outcomes in clinical practice, the mechanisms by which tear pattern, length, and location influence knee joint biomechanics remain poorly understood.

This study developed a finite element model of the knee joint incorporating radial and longitudinal meniscal tears, and simulated the corresponding surgical techniques to analyze the combined effects of tear morphology and repair strategy on stress distribution, contact pressure, hoop stress, and displacement under static loading conditions. The objectives were to: (1) investigate the influence of tear type and size on knee joint stress distribution; and (2) evaluate the biomechanical restoration achieved by different repair techniques following meniscal injury.

The novelty of this study lies in the establishment of a unified finite element analysis framework to systematically compare multiple meniscal tear morphologies and repair strategies under identical static loading conditions. In contrast to previous studies that typically focused on a single tear type or isolated mechanical parameters, the present study integrates tear type, tear length, tear location (red, red-white, and white zones), and surgical approach (repair versus partial meniscectomy) into a single modeling framework, allowing for the simultaneous evaluation of their combined effects on stress distribution, contact pressure, hoop stress, and displacement. On this basis, by comparing different tear morphologies and their corresponding surgical procedures, this study aims to provide a more comprehensive understanding of the mechanical correlations between tear characteristics and repair strategies. This approach offers a new quantitative assessment perspective for investigating the mechanical behavior of torn menisci and provides a more reliable theoretical basis for clinical surgical selection and individualized treatment planning.

## 2 Materials and methods

Imaging data were obtained from a healthy male volunteer (22 years old, height 170 cm, BMI 23 kg/m^2^) with no history of medical or surgical diseases, nor any prior knee joint injury or surgery. Physical examination and X-ray confirmed the absence of knee joint pathology. All methods in this study were carried out in accordance with the Helsinki Declaration. The experimental protocols of this study were approved by the Ethics Committee of Tianjin University Hospital, Tianjin, China (IRB: 2024 Medical Ethics Bureau Pre-020).

### 2.1 Data acquisition

The right knee was scanned using computed tomography (CT) (GE Discovery CT 750 HD) with a slice thickness of 0.625 mm to reconstruct the bony structures, including the femur, tibia, fibula, and patella. Magnetic resonance imaging (MRI) (SIGNA 3.0T; GE, Las Vegas, NV, USA) of the same knee was performed using a 3.0 T scanner with a slice thickness of 1 mm, yielding 250 MRI slices for segmentation of the soft tissues, including the menisci, ligaments, and articular cartilage. Since the CT and MRI scans were performed separately, a rigid registration protocol was applied to align the two imaging datasets. The volunteer was positioned in the supine position with the knee in full extension, and the same fixation support frame was used to minimize positional differences between the two scans.

### 2.2 Three-dimensional reconstruction of the knee joint

The DICOM images acquired from CT and MRI of the knee joint were imported into MIMICS software (Materialise, Leuven, Belgium) for three-dimensional reconstruction of bone and soft tissues. Segmentation of anatomical structures was performed based on grayscale values: the femur, tibia, fibula, and patella were segmented from CT images, while the articular cartilage (femoral, tibial, fibular, and patellar cartilage), menisci (medial and lateral), ligaments (anterior cruciate ligament (ACL), posterior cruciate ligament (PCL), medial collateral ligament (MCL), and lateral collateral ligament (LCL)), and quadriceps tendon were segmented from MRI images.

**FIGURE 1:**
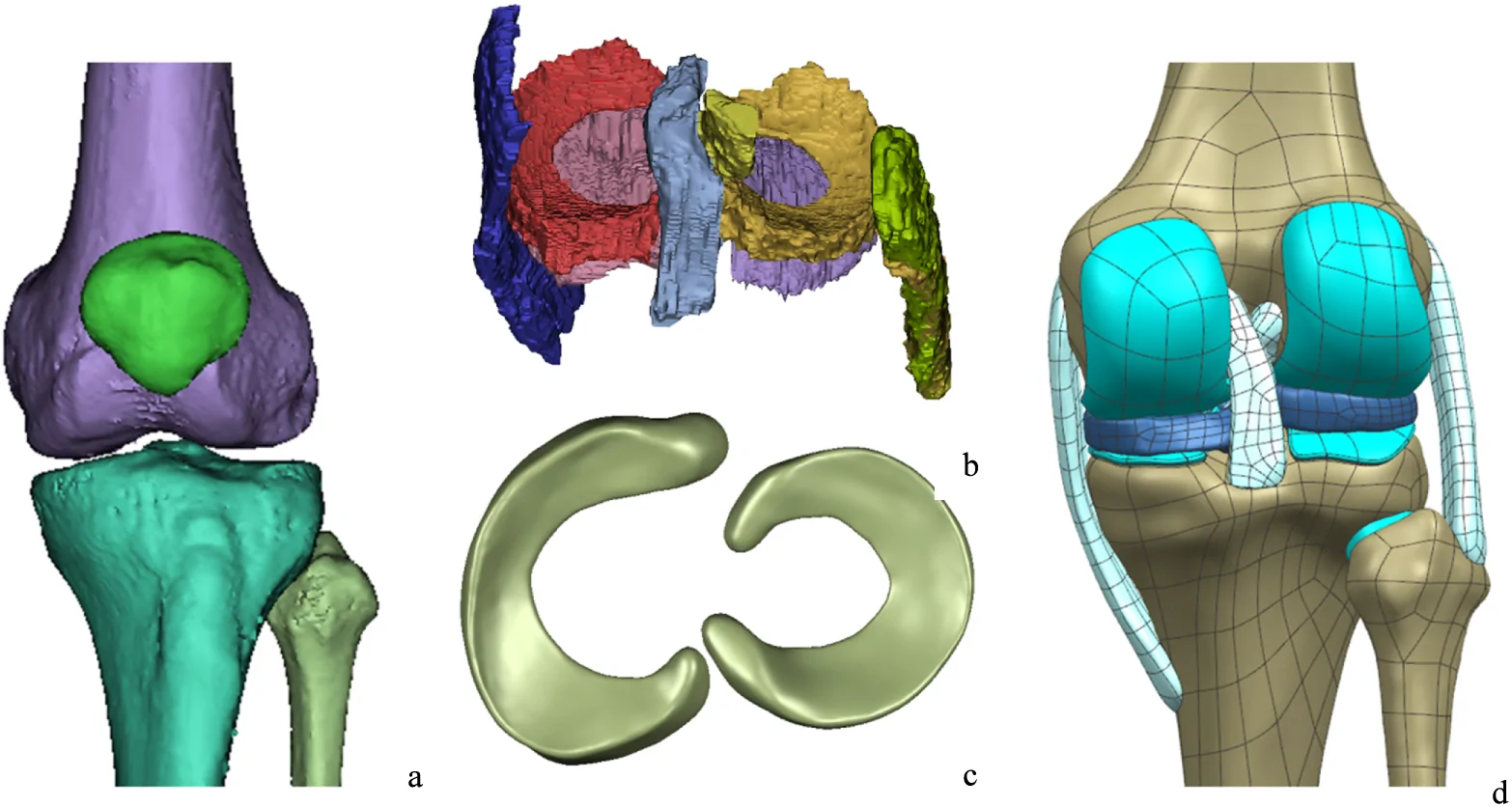
Schematic diagram of the creation of a 3D model of the knee joint. **(a)** Bone tissue extraction; **(b)** Soft tissue extraction; **(c)** Surface smoothing; **(d)** 3D model of the knee joint.

To achieve precise registration between the CT and MRI three-dimensional models, the process was jointly guided by an experienced radiologist and an orthopedic clinician. Anatomical landmarks, including the centers of the medial and lateral femoral condyles, the tibial eminences, and the axes of the distal femur and proximal tibia, were manually identified in both CT and MRI images for initial alignment. Based on these landmarks, the CT bony model and the MRI soft tissue model were aligned through translation and rotation. After registration, two physicians independently inspected and confirmed the consistency of the bony contours and soft tissue positions. If any positional inaccuracies were observed, fine manual adjustments were performed. The workflow for establishing the three-dimensional knee model is illustrated in Figure 1.

### 2.3 Finite element modeling of the knee joint

The three-dimensional geometric model of the knee joint was imported into Abaqus (SIMULIA, Providence, RI, USA) for finite element analysis. Material properties were assigned, meshes were generated, and boundary conditions were defined to establish a complete finite element model of the knee joint. A mesh convergence study was performed to verify mesh independence. The mesh size was considered acceptable if the peak contact pressure error of the tibial cartilage was within 2% compared to a finer reference mesh. Based on this criterion, the final model employed a mesh size of 2.5 mm for bony structures and 1 mm for soft tissues, resulting in 773,394 tetrahedral elements and 201,499 nodes.

### 2.4 Material property assignment

The material parameters of the finite element model of bone structures (femur, tibia, fibula and patella) are shown in Table 1. Cortical bone and cancellous bone are both isotropic elastic materials. The articular cartilage is simulated using the Yeoh model to simulate the isotropic, hyperelastic, and incompressible material behavior (Robinson et al., 2016). The strain energy function of its model is shown below (Equation 1):

$$\Phi =C_{10}\left({\bar{I}}_{1}-3\right)+C_{20}{\left({\bar{I}}_{1}-3\right)}^{2}+C_{30}{\left({\bar{I}}_{1}-3\right)}^{3}+\frac{1}{D_{1}}{\left(J-1\right)}^{2}+\frac{1}{D_{2}}{\left(J-1\right)}^{4}+\frac{1}{D_{3}}{\left(J-1\right)}^{6}$$ (1)

**TABLE 1:** Bone structure material parameters.

References	$E\left(MPa\right)$	ν
Cortical bone (Wang et al., 2021b)	17,000	0.3
Cancellous bone (Du et al., 2022)	400	0.3

Where: $\bar{I_{1}}$ - the first invariant of the modified left Cauchy–Green tensor; J - the elastic volume ratio; $C_{10}$ , $C_{20}$ , $C_{30}$ , $D_{1}$ , $D_{2}$ , $D_{3}$ - independent Yeoh material constants. The values of the parameters $C_{10}$ , $C_{20}$ for normal and degenerated knee cartilage were taken from an experimental study (Robinson et al., 2016),see Table 2. However, the constant $C_{30}$ was assumed to be zero due to a lack of experimental data (Daszkiewicz and Luczkiewicz, 2021). The values of the parameters $D_{1}$ were calculated from the values of $C_{10}$ Poisson’s ratio of ν=0.45 (Table 2).

**TABLE 2:** Articular cartilage material parameters (Robinson et al., 2016).

Parameter	Femoral cartilage	Tibial cartilage
$C_{10}\left(MPa\right)$	1.4	2.0
$C_{20}\left(MPa\right)$	3.6	4.5
$D_{1}\left({MPa}^{-1}\right)$	0.0739	0.0517

Meniscus material properties were defined based on constructed as transversely isotropic hyperelastic material (Gasser et al., 2006), by combining the strain energy density function with the Holzapfel-Gasser-Ogden (HGO) material model, Meniscus material model with the function constructed in the form (Xu et al., 2022), is given as follows Equations 2, 3:

$$U=C_{10}\left({\bar{I}}_{1}-3\right)+\frac{1}{D_{1}}\left(\frac{{\left(J_{el}\right)}^{2}-1}{2}-\text{In}J_{el}\right)+\frac{k_{1}}{2k_{2}}\left(\exp\left[k_{2}{\langle {\bar{E}}_{a}\rangle }^{2}\right]-1\right)$$ (2)

$${\bar{E}}_{\alpha }=\kappa \left({\bar{I}}_{1}-3\right)+\left(1-3\kappa \right)\left({\bar{I}}_{4\left(\alpha \alpha \right)}-1\right)$$ (3)

Where: ${\bar{I}}_{4\left(\alpha \alpha \right)}$ - a pseudoinvariant of the symmetrically modified Cauchy-Green strain tensor, which simulates hard elastic collagen fibers. Among the parameters, $C_{10}$ , $D_{1}$ , $k_{1}$ , $k_{2}$ and κ (Table 3) are used by Abaqus software to simulate real hyperelastic material properties in the calculation.

**TABLE 3:** Meniscus material parameters (Danso et al., 2015; Xu et al., 2022).

Component	$C_{10}\left(MPa\right)$	$D_{1}\left(MPa^{-1}\right)$	$k_{1}$	$k_{2}$	κ
Medial meniscus	1	5e^−3^	5.0	0.9	0
Lateral meniscus	1	5e^−3^	8.5	1.6	0

$C_{10}$ and $D_{1}$ : neo-Hookean constants; $k_{1}$ 和 $k_{2}$ : HGO coefficients; κ : Fiber dispersion and orientation level.

Ligaments material properties were defined based on established nearly incompressible, nonlinear, hyperelastic, transversely isotropic fiber material, with its strain energy function given by Equation 4 (Luczkiewicz et al., 2016):

$$\Phi =C_{1}\left({\bar{I}}_{1}-3\right)+\frac{1}{D_{1}}{\left(J-1\right)}^{2}+F_{2}\left(\lambda \right),J=\det F,{\bar{I}}_{1}=\text{tr}\bar{B}$$ (4)

Contains the Neo-Hookean formula, Two of the constants $C_{1}$ , and $D_{1}$ (Table 4) defines the matrix substance and transverse part $F_{2}\left(\lambda \right)$ according to the stiffness of collagen fibers, and the function $F_{2}\left(\lambda \right)$ satisfies the conditions (Equation 5):

$$\lambda \frac{\partial F_{2}\left(\lambda \right)}{\partial \lambda }=\left\{\begin{array}{ll} 0, & \lambda \leq 1,\\ C_{3}\left(\exp\left(C_{4}\left(\lambda -1\right)\right)-1\right), & 1<\lambda <\lambda^{*},\\ C_{5}\lambda +C_{6}, & \lambda \geq \lambda^{*}, \end{array}\right.$$ (5)

**TABLE 4:** Ligament material parameters (Luczkiewicz et al., 2016).

Component	$C_{1}\left(MPa\right)$	$D_{1}\left(MPa^{-1}\right)$	$C_{3}\left(MPa\right)$	$C_{4}$	$C_{5}\left(MPa\right)$	$\lambda^{*}$
ACL	1.95	0.01366	0.0139	116.22	535.039	1.046
LCL	1.44	0.00252	0.57	48.0	467.1	1.062
MCL	1.44	0.00252	0.57	48.0	467.1	1.062
PCL	3.25	0.0082	0.1196	87.178	431.063	1.035

Constants $C_{1}$ and $D_{1}$ -Neo-Hookean constants, $C_{3}$ - the exponential stress, $C_{4}$ - the rate of collagen uncramping $C_{5}$ - the elastic modulus of the straightened collagen fibers, $\lambda^{*}$ - predetermined fiber stretch value beyond which collagen fibers straighten.

Where λ is the fiber stretch, $\lambda^{*}$ is the limit value of stretch corresponding to straightened fiber, $C_{1}$ is related to the shear modulus $u\left(C_{1}=2/u\right)$ , J is the determinant of the deformation tensor F , $\bar{I_{1}}$ is the first invariant of the deviatoric left Cauchy-Green tensor $\bar{I_{1}}=tr{\bar{FF}}^{T}$ with a deformation gradient of $\bar{F}\left(\bar{F}=J_{F}^{-0.33}F\right)$ .

The stress in the fiber depends on the fiber stretch λ , which is determined by the deformed fiber orientation $a_{d}$ , the deformation gradient F , and the initial fiber orientation $a_{0}\left(\lambda a_{d}=Fa_{0}\right)$ . If the fiber is under compression λ≤1 , it will not be subjected to any compressive stress. As the fiber is stretched between 1 and a predefined value ( $\lambda^{*}$ ), the stiffness of the fiber increases exponentially, beyond which the fiber straightens and the stiffness increases linearly.

Where: $C_{3}$ - the exponential stress; $C_{4}$ - the rate of collagen uncramping; $C_{5}$ - the elastic modulus of the straightened collagen fibers; $C_{6}$ - calculated from the continuity condition of λ , the constant $C_{6}$ is introduced to ensure that the stresses are continuous at $\lambda *\left[C_{6}=\left(e^{C_{4}\left(\lambda *-1\right)}-1\right)\cdot C_{3}-\left(C_{5}\lambda^{*}\right)\right]$ (Li et al., 2020).

### 2.5 Modeling of meniscal tears, partial meniscectomy, and repair

An intact medial meniscus model was first established. Given that the medial meniscus has a significantly higher injury rate than the lateral meniscus and that its posterior horn bears greater load, all tears were simulated in the posterior region of the medial meniscus. Based on previously defined criteria (Kedgley et al., 2019), stable radial tears were defined as those extending less than one-third of the meniscal width, and stable vertical longitudinal tears as those shorter than 10 mm total of 21 meniscal models were developed, including:

Intact medial meniscus (Intact).

Stable and unstable radial tears (SR, UR).

Stable longitudinal tears in the white, red-white, and red zones (SL_W, SL_RW, SL_R).

Unstable longitudinal tears in the white, red-white, and red zones (UL_W, UL_RW, UL_R).

Partial meniscectomy models for stable and unstable radial tears (SR_M, UR_M).

Repair models for stable and unstable radial tears (SR_R, UR_R).

Repair models for stable longitudinal tears in the white, red-white, and red zones (SL_W_R, SL_RW_R, SL_R_R).

Partial meniscectomy model for stable longitudinal tears in the white zone (SL_W_M).

Repair models for unstable longitudinal tears in the white, red-white, and red zones (UL_W_R, UL_RW_R, UL_R_R).

Partial meniscectomy model for unstable longitudinal tears in the white zone (UL_W_M).

Partial meniscectomy models were created using SolidWorks, and meniscal repair (suturing) was simulated using the “Tie” constraint in Abaqus. The peak contact pressure, von Mises stress, hoop stress, and compressive displacement were compared across all tear, repair, and meniscectomy models.

### 2.6 Loading and boundary conditions

The boundary conditions of this study are defined as follows: the lower ends of the tibia and fibula are completely fixed, the femur is free in the axial direction, varus and valgus angles, and internal and external rotation angles, the contact between the components of the knee joint is frictional contact, and the ligaments and bones are rigidly connected. Tangential sliding with a friction coefficient of 0.02 is set between cartilages and between cartilages and menisci (Daszkiewicz and Luczkiewicz, 2021), and the cartilages and bone tissues and ligaments and bone tissues are rigidly connected. This study employed a static mechanical simulation. To simulate the knee joint loading during single-leg standing, an axial load of 1150 N was applied to the proximal end of the femur, following the protocols reported by Zhang et al. (2021) and Zhang et al. (2019).

### 2.7 Establishment of the cadaveric biomechanical experiment

A cadaveric knee specimen exhibiting normal mechanical alignment was first prepared, and biomechanical testing was subsequently performed according to the designed loading protocol. The experimental protocol was approved by the Medical Ethics Committee of the Second Hospital of Tangshan (IRB: (2026) Medical Ethics Bureau Pre-15).

A fresh-frozen knee specimen (including the femur, tibia, and fibula) was obtained from a young male donor (height: 173 cm; body weight: 72 kg; BMI: 24.1), whose anthropometric parameters closely matched those of the volunteer used for finite element modeling. Based on the specimen data, the following morphological parameters were measured: femoral condylar AP dimension (63.65 mm) and ML dimension (72.92 mm), and tibial AP dimension (56.76 mm) and ML dimension (75.71 mm). The specimen was pre-screened to exclude trauma, deformity, and other pathological conditions. Specimen preparation was performed by a specialist orthopedic surgeon. The skin surrounding the knee joint was initially removed. The muscles of the thigh and lower leg were then meticulously dissected to achieve smooth bone surfaces for mounting, while preserving the mid-to-proximal femoral shaft and the mid-portion of the tibia and fibula for subsequent fixture attachment. The medial and lateral collateral ligaments, the anterior and posterior cruciate ligaments, and the patellar ligament were carefully retained. Finally, the posterior joint capsule was incised to facilitate the insertion of Fuji prescale pressure-sensitive film for contact data acquisition.

**FIGURE 2:**
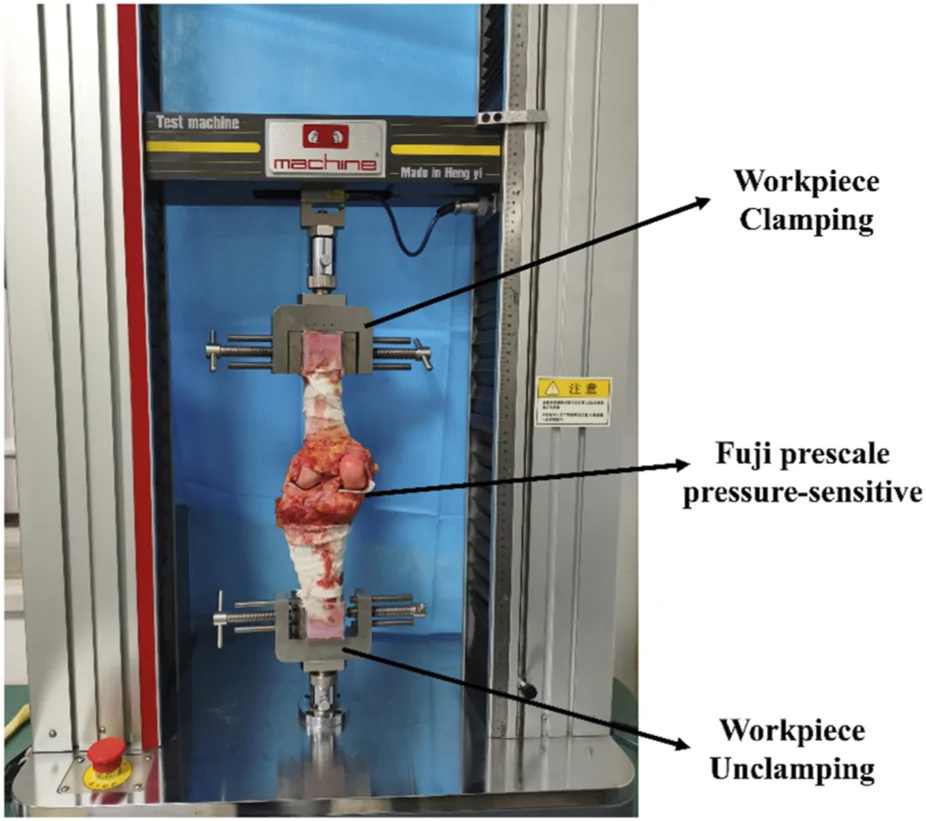
Knee cadaver experiment.

The prepared specimen was mounted on a universal mechanical testing machine using customized fixtures, and the machine height was adjusted to accommodate the specimen. Fuji prescale pressure-sensitive film was trimmed to dimensions slightly exceeding the margins of the medial and lateral tibiofemoral compartments and inserted into the joint spaces to record contact area data. After installation, the knee was positioned in neutral alignment at 0° of flexion, and vertical compressive loading was applied with a target load of 1150 N. The experimental setup is illustrated in Figure 2. An initial preload of 20 N was first applied to secure the specimen, eliminate the joint gap, and establish initial articular contact. The load was then progressively increased at a rate of 20 N/s until reaching the target load of 1150 N, which was maintained for 1 minute prior to unloading. The Fuji pressure-sensitive film was subsequently retrieved and allowed to rest for 20 min before data extraction. The contact area and contact region distribution were subsequently analyzed.

## 3 Results

### 3.1 Verification of the validity of the finite element model

**FIGURE 3:**
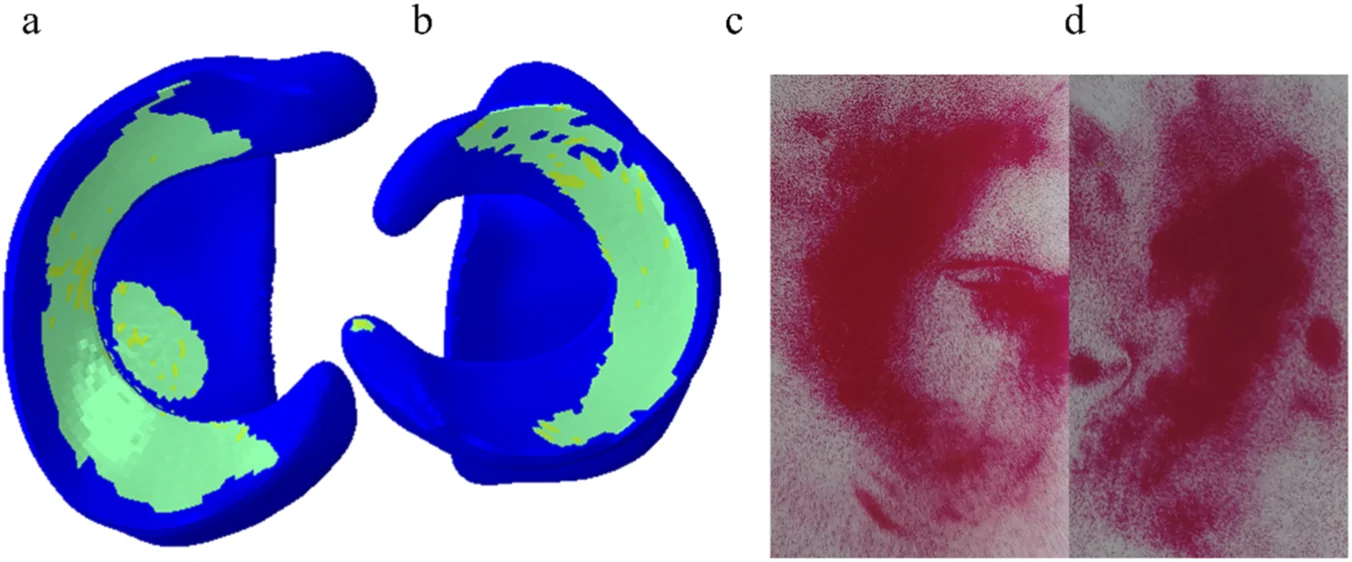
Contact distribution maps of the tibiofemoral compartments. **(a)** Contact distribution of the medial compartment predicted by the finite element model; **(b)** Contact distribution of the lateral compartment predicted by the finite element model; **(c)** Contact distribution of the medial compartment obtained from the biomechanical experiment; **(d)** Contact distribution of the lateral compartment obtained from the biomechanical experiment.

Due to limitations of the experimental equipment, direct measurement of contact pressure was not feasible in this study. Therefore, a dual validation approach was adopted, combining direct contact area verification with literature-based contact pressure comparison. Figure 3 presents the compartment contact area maps of the knee joint under a static axial load of 1150 N, obtained from both the biomechanical experiment and the finite element model. Quantitative analysis revealed that the medial compartment contact area predicted by the finite element model was 535.51 mm^2^ (Figure 3a), accounting for 41.77% of the total medial compartment area. In comparison, the medial compartment contact area measured from the biomechanical cadaveric experiment was 567.98 ± 33.43 mm^2^ (Figure 3c), representing 41.81% of the total medial compartment area. The discrepancy in medial compartment contact area between the biomechanical experiment and the finite element model was 5.72%. It should be noted that the contact regions predicted by the finite element model (Figures 3a,b) and those measured by pressure-sensitive films (Figures 3c,d) exhibited certain differences in spatial distribution. These discrepancies were primarily attributed to the geometric unfolding effect of the pressure-sensitive films and inter-specimen variability. Despite these differences, the validation results for contact area and peak pressure remained consistent in overall trends. Therefore, the biomechanical experiments provided preliminary validation of the knee finite element model. In addition, the peak pressures predicted by the finite element model for the medial and lateral compartments fell within the ranges reported in the literature (Thambyah, 2007; Wang J. Y. et al., 2021; Zhang et al., 2021; Xu et al., 2022; Yang et al., 2023). These findings collectively confirm the validity of the neutral-position normal knee finite element model established in this study.

### 3.2 Peak contact pressure and mises stress of the knee joint

#### 3.2.1 Intact and torn menisci

**FIGURE 4:**
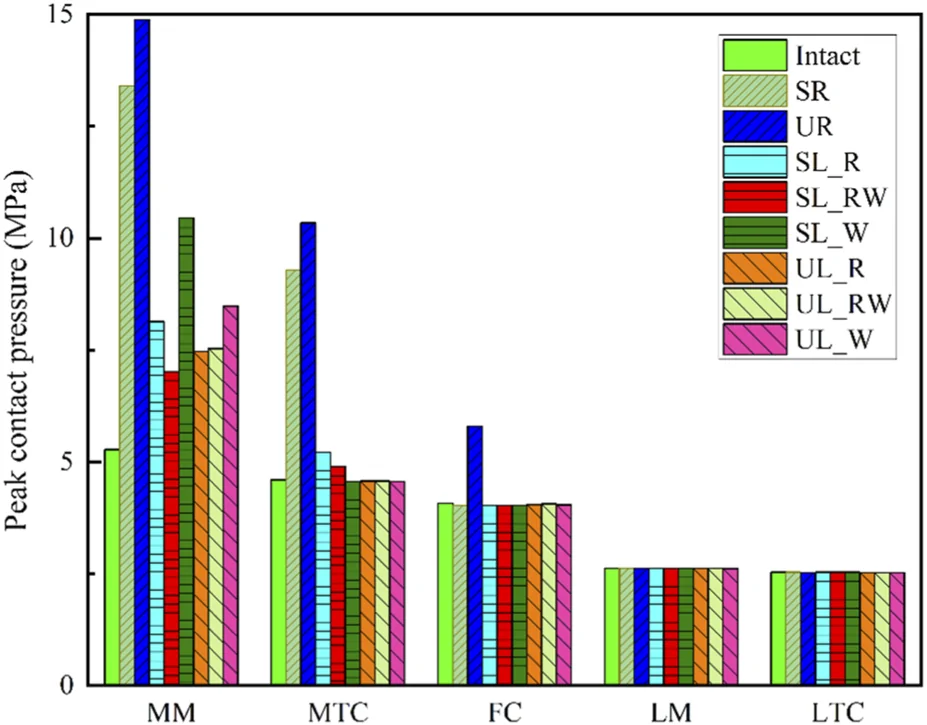
Peak contact pressure of the intact and torn menisci. MM: Medial meniscus MTC: Medial tibial cartilage FC: Femoral cartilage LM: Lateral meniscus LTC: Lateral tibial cartilage SR: Stable radial tears UR: Unstable radial tears SL_R: Stable longitudinal tears and red zones SL_RW: Stable longitudinal tears in the red-white SL_W: Stable longitudinal tears in the white UL_R: Unstable longitudinal tears in the red zones UL_RW: Unstable longitudinal tears in the red-whit UL_W: Unstable longitudinal tears in the white.

The peak contact pressurees of the knee joint components under static loading, comparing intact and torn meniscus conditions, are illustrated in Figure 4. Relative to the intact medial meniscus (5.28 MPa), all tear configurations resulted in increased peak contact pressure within the medial compartment. Radial tears consistently induced higher peak stresses than longitudinal tears, with unstable radial tears demonstrating the greatest elevation (14.88 MPa), corresponding to a 181.82% increase.

Furthermore, radial tears significantly elevated the peak contact pressure of the medial tibial cartilage. Within the radial tear subset, stable tears confined to the white zone exhibited the highest peak contact pressure in the medial meniscus (10.45 MPa), representing a 97.92% increase over the intact state. In contrast, the lateral compartment (lateral meniscus and lateral tibial cartilage) remained largely unaffected by medial meniscal tears.

#### 3.2.2 Models of radial meniscal tear, repair, and partial meniscectomy

**FIGURE 5:**
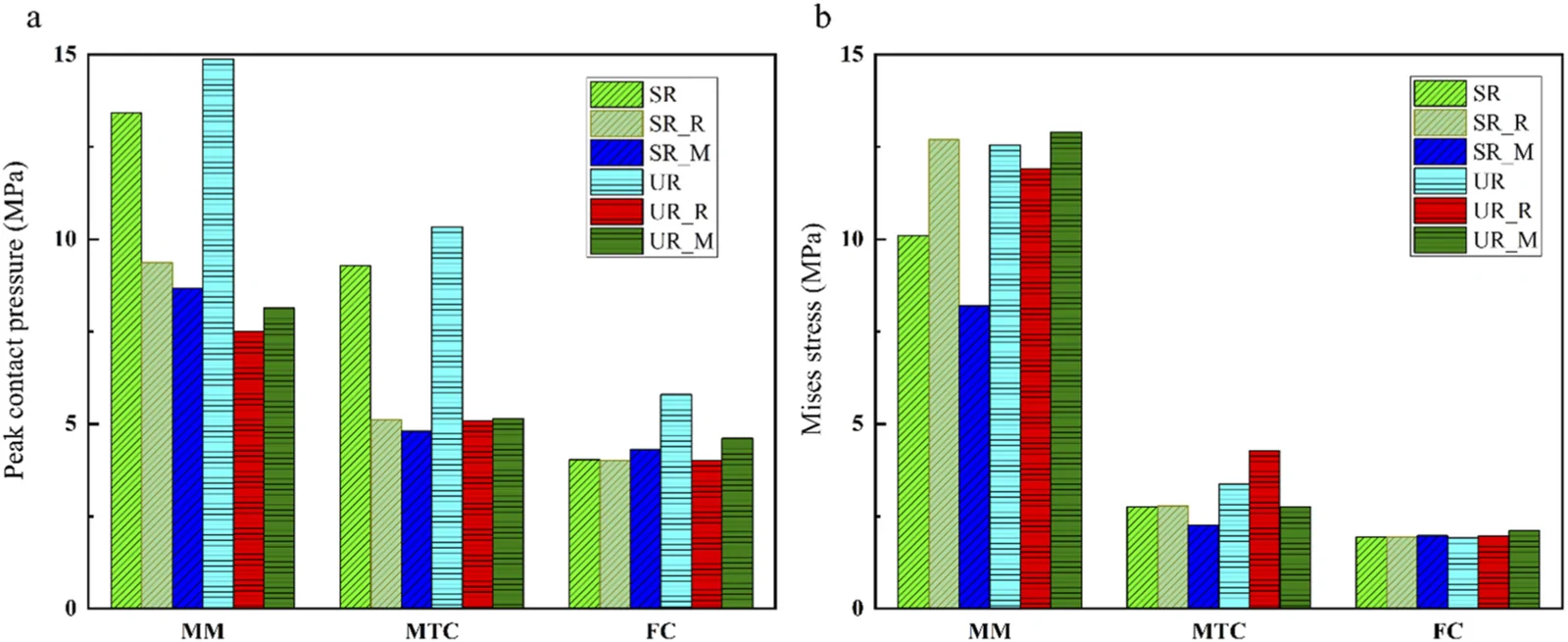
Stresses in knee joint components following radial meniscal tear, repair, and partial meniscectomy. **(a)** Peak contact pressure; **(b)** Mises stress; MM: Medial meniscus MTC: Medial tibial cartilage FC: Femoral cartilage SR: Stable radial tears SR_R: Repair models for stable radial tears SR_M: Partial meniscectomy models for stable and unstable radial tears UR: Unstable radial tears UR_R: Repair models for unstable radial tears UR_M: Partial meniscectomy models for stable and unstable radial tears.

Figure 5 presents the peak contact and von Mises stresses of knee joint components following radial meniscal tear, repair, and partial meniscectomy under static loading. Both repair and partial meniscectomy effectively reduced the peak contact pressure of the medial meniscus and tibial cartilage, with greater reductions observed for unstable radial tears than for stable ones.

For stable radial tears, partial meniscectomy markedly decreased the peak contact pressure of the meniscus (Table 5). However, no marked difference was found between the two surgical techniques in terms of peak contact pressure reduction for the tibial or femoral cartilage. In contrast, for unstable radial tears, meniscal repair resulted in a marked reduction in meniscal peak contact pressure (Table 5), whereas partial meniscectomy showed a lesser effect.

**TABLE 5:** Peak contact stresses in each component of the knee joint following meniscal repair and partial meniscectomy for radial tears, and their percentage changes relative to the torn state.

Component	SR	SR_R	Percentage change (%)	SR_M	Percentage change (%)	UR	UR_R	Percentage change (%)	UR_M	Percentage change (%)
MM	13.41	9.36	−30.20	8.67	−35.35	14.88	7.50	−49.60	8.13	−45.36
MTC	9.29	5.11	−45.00	4.81	−44.99	10.34	5.09	−50.77	5.15	−50.19
FC	4.03	4.01	−0.50	4.31	−0.50	5.80	4.01	−30.86	4.61	−20.52

MM: Medial meniscus MTC: Medial tibial cartilage FC: femoral cartilage.

SR: Stable radial tears SR_R: repair models for stable radial tears.

SR_M: partial meniscectomy models for stable and unstable radial tears.

UR: Unstable radial tears UR_R: repair models for unstable radial tears.

UR_M: partial meniscectomy models for stable and unstable radial tears.

Regarding von Mises stress, partial meniscectomy markedly reduced values in stable radial tears, while neither repair nor partial meniscectomy had appreciable effects on von Mises stress in unstable radial tears.

#### 3.2.3 Models of stable longitudinal meniscal tear, repair, and partial meniscectomy

**FIGURE 6:**
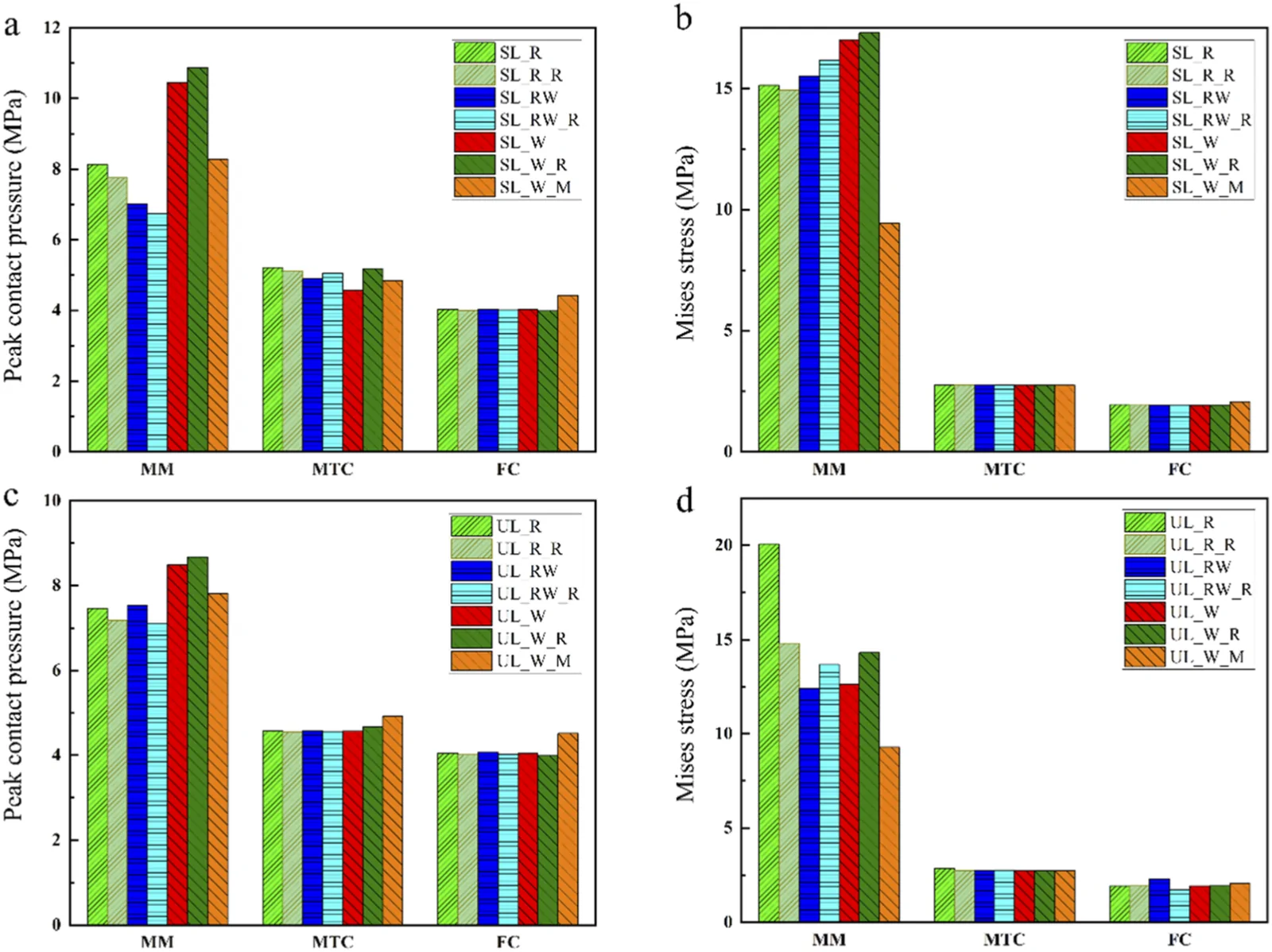
Stresses in knee joint components following longitudinal meniscal tear, repair, and partial meniscectomy. **(a)** Stable longitudinal tear peak contact pressure; **(b)** Stable longitudinal tear mis-stress; **(c)** Unstable longitudinal tear peak contact pressure; **(d)** Unstable longitudinal tear mis-stress.

Figure 6 presents the peak contact and von Mises stresses of knee joint components following longitudinal meniscal tear, repair, and partial meniscectomy under static loading. For stable longitudinal tears (Figure 6a), meniscal repair reduced the peak contact pressure of the meniscus by 4.55% and 3.85% for tears in the red and red-white zones, respectively. In contrast, for stable tears located in the white zone, repair increased the peak contact pressure, whereas partial meniscectomy markedly reduced it by 2.17 MPa (20.77%). Partial meniscectomy also substantially decreased von Mises stress by 7.58 MPa (44.56%) in this region (Figure 6b).

For unstable longitudinal tears (Figure 6c), meniscal repair had a minimal effect on peak contact pressure in the red and red-white zones. However, for unstable tears in the white zone, partial meniscectomy appreciably reduced peak contact pressure by 0.67 MPa (7.90%) and von Mises stress by 3.36 MPa (26.58%). Regardless of tear length or location, neither meniscectomy nor partial meniscectomy appreciably affected the peak contact or von Mises stresses of the medial tibial or femoral cartilages.

### 3.3 Hoop stress

#### 3.3.1 Radial meniscal tear

**FIGURE 7:**
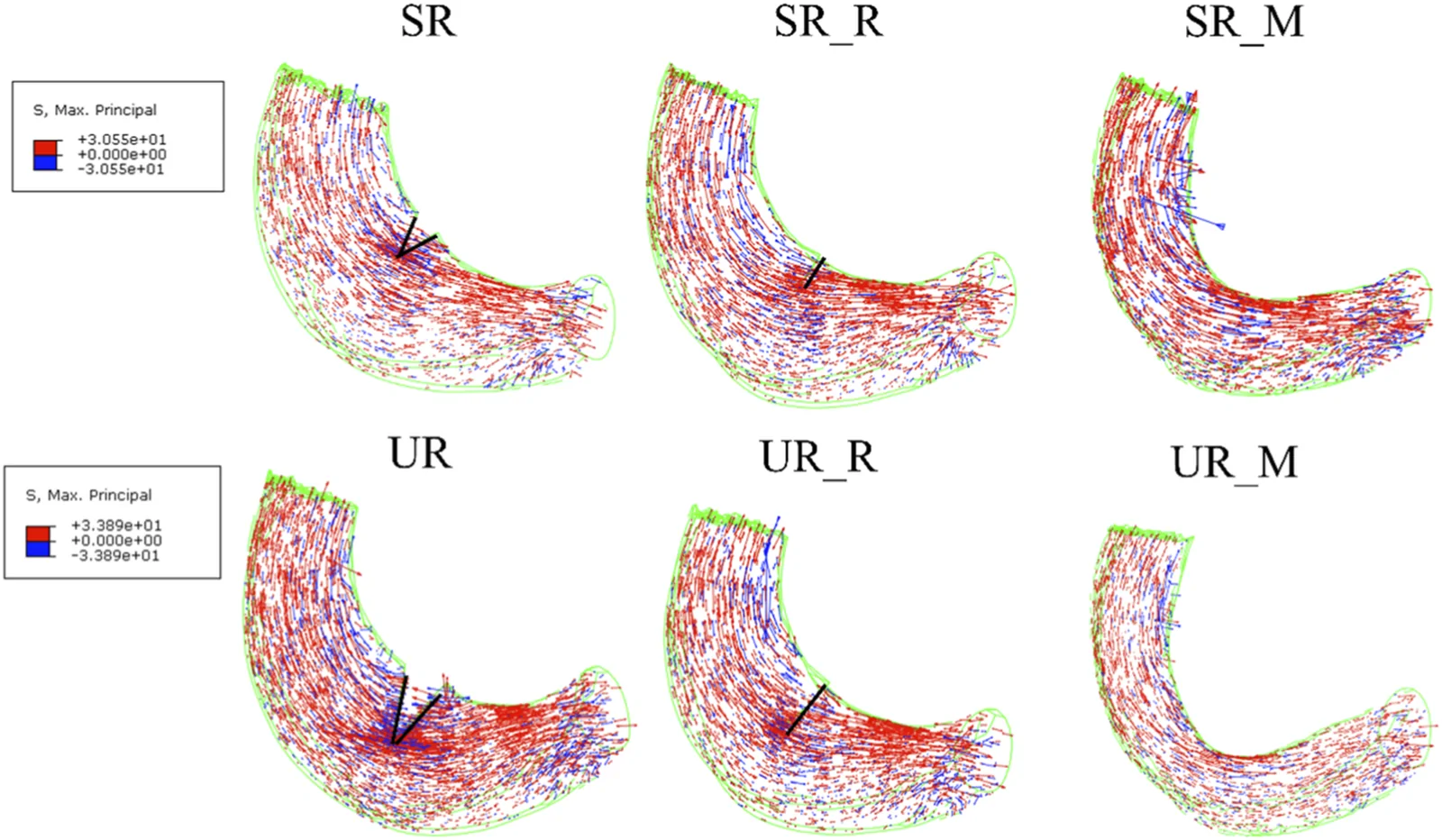
Schematic diagram of radial tear hoop stress. In the diagram, the blue arrows indicate the direction of hoop stress, the red arrows indicate tensile stress, and the blue arrows indicate compressive stress.

For radial meniscal tears of varying lengths, both repair and partial meniscectomy restored hoop stress transmission and alleviated stress concentration. Partial meniscectomy resulted in a marked reduction in hoop stress, as illustrated in Figure 7. In stable radial tears, partial meniscectomy decreased hoop stress by 9.66 MPa, compared to a 2.03 MPa reduction with repair. For unstable radial tears, the reduction was 18.31 MPa with partial meniscectomy and 6.70 MPa with repair.

Radial tears disrupt the hoop collagen fibers and reduce the effective cross-sectional area available for load transmission, leading to stress concentration at the tear apex. The magnitude of stress concentration was greater for unstable than for stable radial tears. Additionally, the reversal of hoop stress direction at the tear site may provide a mechanistic explanation for the propagation of radial tears.

#### 3.3.2 Longitudinal meniscal tear

**FIGURE 8:**
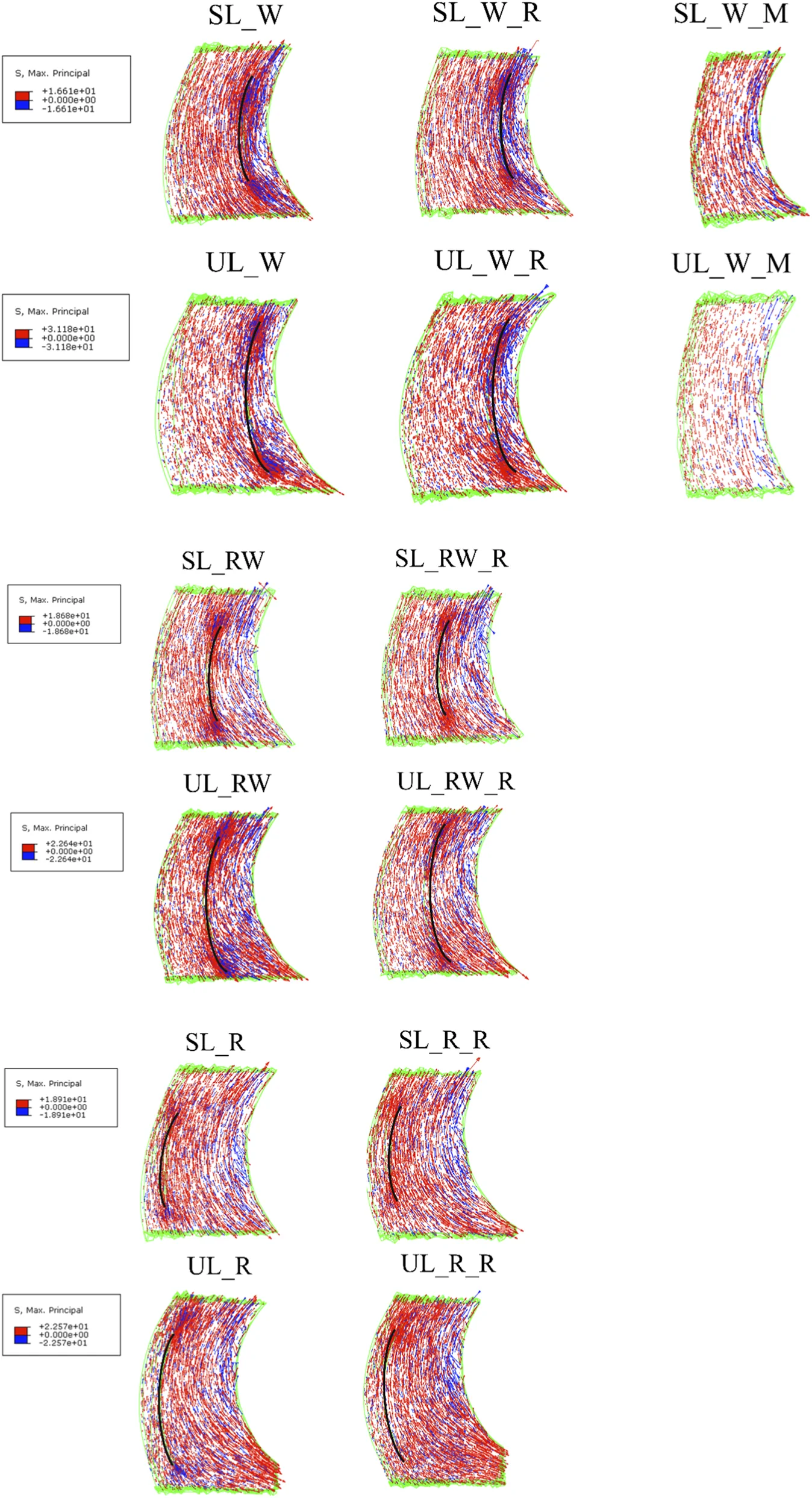
Schematic diagram of hoop stress in longitudinal tears across different zones. In the diagram, the blue arrows indicate the direction of hoop stress, the red arrows indicate tensile stress, and the blue arrows indicate compressive stress.

Compared with radial tears, longitudinal tears exhibited lower peak hoop stress and a more uniform stress distribution. The effects of repair and partial meniscectomy varied by tear location (Figure 8). For stable longitudinal tears in the white zone, neither repair nor partial meniscectomy appreciably altered the hoop stress around the tear. In contrast, for unstable longitudinal tears in the white zone, both surgical techniques markedly reduced hoop stress. For longitudinal tears located in the red or red-white zones, repair resulted in a substantial decrease in hoop stress. Moreover, repair of unstable longitudinal tears was more effective than that of stable tears in alleviating stress concentration and restoring hoop stress transmission.

### 3.4 Meniscal displacement

Under a static axial load of 1150 N, the displacement of the meniscus was markedly higher in unstable radial tears than in stable tears. Meniscal repair effectively constrained lateral extrusion, thereby attenuating overall displacement. Conversely, partial meniscectomy in stable radial tears was associated with increased compressive displacement. For longitudinal tears, repair produced no appreciable change in displacement, whereas partial meniscectomy elicited a notable reduction (Table 6).

**TABLE 6:** Displacement of the meniscus under different tear types and corresponding surgical techniques.

Tear type	Displacement (mm)	Tear type	Displacement (mm)	Tear type	Displacement (mm)
Intact	4.32	SL_R	4.23	UL_R	4.24
SR	4.33	SL_R_R	4.23	UL_R_R	4.25
SR_R	4.27	SL_RW	4.24	UL_RW	4.21
SR_M	4.51	SL_RW_R	4.28	UL_RW_R	4.27
UR	4.43	SL_W	4.27	UL_W	4.19
UR_R	4.27	SL_W_R	4.27	UL_W_R	4.23
UR_M	4.42	SL_W_M	3.75	UL_ W_M	3.88

## 4 Discussion

The findings of this study highlight the multifactorial nature of meniscal stress distribution, which is modulated by tear types, locations, tear lengths, and the specific surgical intervention employed. Of these factors, the surgical approach—particularly meniscal repair versus partial meniscectomy—exerted a pronounced influence on the restoration of hoop stress. Repair consistently facilitated recovery of physiological stress patterns, whereas the biomechanical outcomes of partial meniscectomy were contingent upon tear morphology.

The present investigation utilized a subject-specific finite element model of the knee joint to evaluate the influence of meniscal tear morphology (stable vs. unstable radial and longitudinal tears) and subsequent surgical interventions (repair and partial meniscectomy) on stress distribution under static axial loading. Prior to analyzing pathological conditions, the fidelity of the model was assessed by comparing predictions for the intact knee with experimental data obtained from cadaveric knee specimen testing. The discrepancy in medial compartment contact area between the biomechanical experiment and the finite element model was 5.72%, and as shown in Figures 3a,c), the contact regions exhibited satisfactory consistency between the two approaches. The computed peak contact pressures in the medial and lateral compartments were 5.28 MPa and 4.26 MPa, respectively, which demonstrated excellent agreement with previously published biomechanical measurements of intra-articular pressure obtained from cadaveric knee specimens and finite element simulations of intact menisci (Thambyah, 2007; Allaire et al., 2008; Willinger et al., 2020; Wang J. Y. et al., 2021; Zhang et al., 2021; Xu et al., 2022; Yang et al., 2023). This concordance substantiates the capacity of the developed model to reliably represent the biomechanical behavior of the knee joint, thereby establishing a credible foundation for investigating the mechanical consequences of meniscal tears and their surgical management.

Evaluation of radial meniscal tears demonstrated stress concentration at the tear apex, corroborating the observations of Dong et al. (2014) and Zhang et al. (2019). This biomechanical response is attributable to interruption of the hoop collagen fibers, which compromises the transmission of hoop stress. Notably, unstable radial tears exhibited both higher magnitude and reversed direction of hoop stress relative to stable tears, a configuration that may facilitate bidirectional tear propagation and enlargement of the tear gap—findings that align with the clinical progression of meniscal lesions. Both surgical interventions, namely, repair and partial meniscectomy, attenuated peak contact pressure at the menisco-tibial interface. The effect was more pronounced in stable tears, with partial meniscectomy conferring a greater reduction than repair. Additionally, partial meniscectomy resulted in a substantial decrease in hoop stress. From a clinical perspective, radial tears frequently occur in avascular or hypovascular zones with limited healing capacity, rendering partial meniscectomy a commonly employed surgical strategy. The current study provides biomechanical substantiation for this approach by elucidating its capacity to restore favorable stress distributions within the meniscus.

Evaluation of stable and unstable longitudinal meniscal tears demonstrated that the resultant alterations in peak contact pressure were contingent upon tear location and length. Stress elevations were most marked in the white zone, although all tear types induced an increase in meniscal stress. Of note, the magnitude of stress increase was substantially lower for longitudinal tears than for radial tears. This observation corroborates the findings of Kedgley et al. (Kedgley et al., 2019), who established that longitudinal tears, oriented parallel to the hoop collagen fibers, minimally perturb the transmission of hoop stress. Our data substantiate this mechanism (Figure 8), stress concentration was localized to the tear apex, with the surrounding stress field remaining largely intact. From a clinical standpoint, untreated longitudinal tears are susceptible to bidirectional propagation under physiological loading, which may precipitate tear extension and compromise meniscal function over time.

The present study offers unique biomechanical validation for the application of meniscal repair and partial meniscectomy in longitudinal tears, with treatment efficacy contingent upon tear location. In tears involving the red or red-white zones, repair effected a significant reduction in both peak contact and hoop stress. This observation implies that repair partially reinstates the transmission of hoop stress, thereby facilitating the restoration of meniscal function. These biomechanical findings are consonant with established clinical practice, wherein repair is preferentially indicated for tears situated in vascularized regions endowed with enhanced healing capacity. For longitudinal tears confined to the white zone, meniscal repair proved ineffective in reducing peak contact pressure. Conversely, partial meniscectomy elicited substantial reductions in peak contact pressure, von Mises stress, and compressive displacement, with the effect being most pronounced in stable tears. These observations provide biomechanical substantiation for the clinical preference for partial meniscectomy in avascular regions characterized by poor healing potential. The differential surgical management of longitudinal meniscal tears according to anatomical location is underpinned by both regional vascularity and the biomechanical rationale established in the present investigation.

Meniscal tears disrupt the intrinsic structure of the meniscus, compromising its load-bearing capacity under compression. This leads to narrowing of the joint space and elevated peak contact pressure within the knee compartments. The resultant reduction in meniscal structural integrity increases the contact area between the femoral and tibial cartilages, thereby elevating stress on the tibial cartilage. This biomechanical alteration may contribute to the progression of osteoarthritis (Hunter et al., 2006; Wheatley et al., 2015).

As depicted in Figure 4, longitudinal tears initially induce alterations confined to meniscal stress. However, with time, stress elevation extends to the tibial and femoral cartilages. This temporal progression is corroborated by previous case-control studies, which have established that knees harboring meniscal tears even in the absence of concomitant cartilage pathology exhibit an increased susceptibility to osteoarthritis relative to those with intact menisci (Englund et al., 2009; Bian et al., 2025). Collectively, these findings advocate for timely surgical intervention as a prophylactic measure against the development of osteoarthritis.

The findings of this study indicate that meniscal repair and partial meniscectomy are both capable of partially restoring the biomechanical function of the meniscus. Nevertheless, the efficacy of each surgical approach is contingent upon tear morphology, including types, lengths, and locations. The observed variability in biomechanical restoration across techniques highlights the importance of an individualized surgical strategy tailored to the specific features of the meniscal injury.

Although the meniscal tear model established in this study enables a systematic evaluation of the biomechanical responses across different tear types and surgical strategies, it should be noted that the model involves certain simplifications in both the simulation of the injury and the repair mechanism. First, in terms of injury simulation, all tears were created by artificial cutting, which differs fundamentally from naturally occurring pathological tears. Real meniscal tears are typically accompanied by tissue degeneration, fiber disruption, and matrix damage with irregular margins, whereas artificial cuts provide regular and smooth geometric edges that cannot fully capture the heterogeneous material properties of pathological tissue. In addition, pathological tears often involve microdamage in the surrounding tissue, whereas the present model assumes that the tissue adjacent to the tear retains healthy material properties. This may overestimate the residual hoop tension after tearing, and the sharp edges may induce local stress concentrations in finite element analysis. Second, regarding the repair mechanism, surgical repair was modeled using simplified “Tie” constraints in Abaqus, assuming perfect bonding between tissue segments. This approach cannot account for suture elasticity, gap formation, knot loosening, or progressive tissue remodeling and healing over time, and thus may overestimate the immediate mechanical stability achieved by surgical repair. Nevertheless, the primary aim of this study is to compare the relative mechanical differences among various tear types and surgical interventions, rather than to predict absolute stress values. Therefore, the above simplifications remain a valid and reproducible research approach, and the findings provide important reference value for clinical surgical decision-making.

Several limitations should be acknowledged in this study.

1. Due to experimental constraints, the cadaveric experiment was validated solely based on contact area measurements, without experimental verification of strain distribution or peak stress. In addition, the experiment relied on only one specimen, which may not fully represent anatomical variations across populations. Future work should expand the validation metrics and include more specimens to improve the reliability of the conclusions.
2. The finite element model was constructed from imaging data obtained from a single healthy volunteer. Since knee geometry, cartilage thickness, meniscal morphology, and ligament properties vary substantially among individuals, the findings may not be fully representative of the broader patient population. Future studies should develop more subject-specific models to enhance the generalizability of the results.
3. This study only investigated static loading conditions by applying a compressive force of 1150 N. However, meniscal injuries predominantly occur during dynamic activities such as walking, running, and squatting. Dynamic loading generates complex combinations of compression, shear, and rotational forces that cannot be fully reproduced by static simulations. Future studies should incorporate dynamic loading conditions to better reflect the physiological loading of the knee joint.
4. Muscle forces were not incorporated into the finite element model. The stability of the knee joint during movement is significantly influenced by surrounding muscles, including the quadriceps, hamstrings, and gastrocnemius. Ignoring muscle loading may alter the predicted stress distribution within the meniscus and cartilage. Future work should integrate musculoskeletal modeling to more comprehensively analyze the effects of muscle forces on meniscal biomechanics.
5. This study only investigated radial and longitudinal tears located at the posterior horn of the medial meniscus. Other clinically relevant tear types were not considered, which limits the applicability of the findings to the full spectrum of meniscal injuries. Future work should include a wider range of tear morphologies and locations to improve the clinical generalizability of the conclusions.
6. The material properties assigned to the model were largely adopted from previous literature rather than measured directly from the specimen or volunteer. Although this approach is common in finite element modeling, uncertainties in material parameters may still influence stress predictions.
7. This study evaluated only immediate postoperative biomechanics and did not simulate biological healing, tissue remodeling, degeneration, or long-term clinical outcomes. Therefore, although the model can predict stress recovery trends after repair or partial meniscectomy, it cannot directly infer long-term clinical efficacy. In addition, the conclusions are based solely on biomechanical metrics, without support from clinical functional scores, pain assessments, or long-term follow-up data.

## 5 Conclusion

Stress distribution in the meniscus and its components is influenced by tear types, lengths, locations, and surgical technique. Surgical outcomes significantly depend on tear types, lengths, and locations. The extent of biomechanical restoration varies considerably among different surgical techniques, suggesting that clinical procedure selection should be based on personalized consideration of injury characteristics.

## Data Availability

The raw data supporting the conclusions of this article will be made available by the authors, without undue reservation.
